# Supplementary material for: Identification of photoperiod-induced specific miRNAs in the adrenal glands of Sunite sheep (Ovis aries)
Source: Front Vet Sci. 2022 Jul 22;9:888207. doi: 10.3389/fvets.2022.888207 (PMC9354845; doi:10.3389/fvets.2022.888207)
Supplement: Supplementary file 1 [file Data_Sheet_1.ZIP › Supplementary file/miRDeep2 file.zip!/miRDeep2 file/Novel_Structure/LP42A3/Novel_648.pdf]

Provisional ID	:	NC_019473.2_17559
Score total	:	2.8
Score for star read(s)	:	-1.3
Score for read counts	:	0
Score for mfe	:	2.5
Score for randfold	:	1.6
Score for cons. seed	:	
Total read count	:	29
Mature read count	:	29
Loop read count	:	0
Star read count	:	0

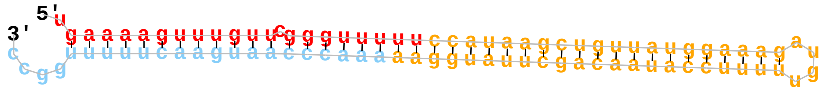

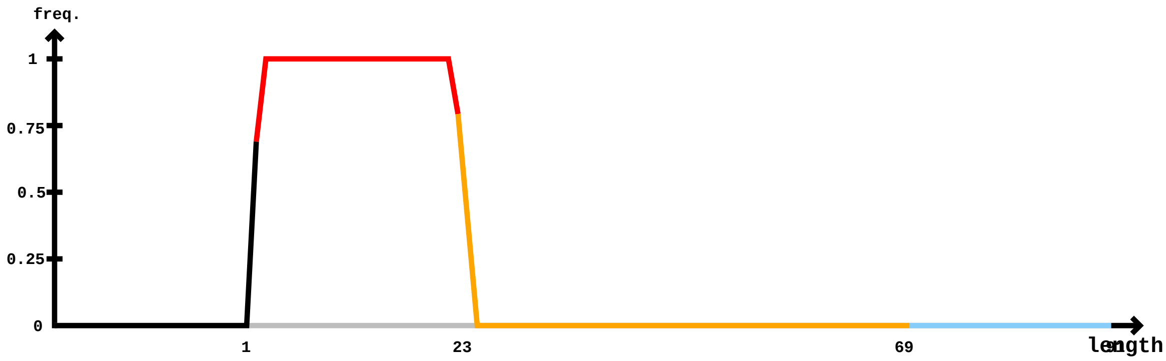

Mature   Star

```
5'- cccacagacacuggguuggugaaaaguuuguucggguuuuuccauaagcuguuauggaaagauguuuuuccauaacagcuuauggaaaaacccaaugaacuuuuuggccaa   -3'    exp
    ((((.......))))(((((.((((((((((.(((((((((((((((((((((((((((((....)))))))))))))))))))))))))))))))))))))))..)))))   reads   mm   sample
    ...................ugaaaaguuuguucggguuuu.......................................................................   2       0    seq
    ...................ugaaaaguuuguucggguuuuu......................................................................   17      0    seq
    ...................ugaaaaguuuguucggguuuuuc.....................................................................   1       0    seq
    ....................Aaaaaguuuguucggguuuu.......................................................................   4       1    seq
    ....................gaaaaguuuguucggguuuuu......................................................................   5       0    seq
```
